# Full scale, microscopically resolved tomographies of sandstone and carbonate rocks augmented by experimental porosity and permeability values

**DOI:** 10.1038/s41597-023-02259-z

**Published:** 2023-06-07

**Authors:** Matheus Esteves Ferreira, Mariana Rodrigues Del Grande, Rodrigo Neumann Barros Ferreira, Ademir Ferreira da Silva, Márcio Nogueira Pereira da Silva, Jaione Tirapu-Azpiroz, Everton Lucas-Oliveira, Arthur Gustavo de Araújo Ferreira, Renato Soares, Christian B. Eckardt, Tito J. Bonagamba, Mathias Steiner

**Affiliations:** 1 IBM Research, Av. República do Chile, 330, Rio de Janeiro, RJ 20031-170 Brazil; 2 University of São Paulo, São Carlos Institute of Physics, São Carlos, 13560-970 Brazil; 3 Solintec Consultoria e Serviços de Geologia Ltda, Rio de Janeiro, 21031-490 Brazil

**Keywords:** Applied physics, Materials science

## Abstract

We report a dataset containing full-scale, 3D images of rock plugs augmented by petrophysical lab characterization data for application in digital rock and capillary network analysis. Specifically, we have acquired microscopically resolved tomography datasets of 18 cylindrical sandstone and carbonate rock samples having lengths of 25.4 mm and diameters of 9.5 mm. Based on the micro-tomography data, we have computed porosity-values for each imaged rock sample. For validating the computed porosity values with a complementary lab method, we have measured porosity for each rock sample by using standard petrophysical characterization techniques. Overall, the tomography-based porosity values agree with the measurement results obtained from the lab, with values ranging from 8% to 30%. In addition, we provide for each rock sample the experimental permeabilities, with values ranging from 0.4 mD to above 5D. This dataset will be essential for establishing, benchmarking, and referencing the relation between porosity and permeability of reservoir rock at pore scale.

## Background & Summary

The use of X-ray micro-computed-tomography (*μ*CT) has transformed the study of porous media such as reservoir rocks. Extracted from high-resolution 3D images, the spatial distribution, geometry, and morphology of the pore space is now being used as a basis for computational fluid dynamics simulations and for estimating physical properties such as porosity and permeability. In rock samples most of the pores have diameters of the order of micrometers or below. However, the rock samples, typically cylindrical in shape and referred to as “plugs”, have a dimension in the centimeter range. As a result, a trade-off exists between the overall sampled volume of the rock plug and the microscopic resolution that can be achieved. Consequently, the literature predominantly reports either low-resolution, i.e. 10–100 *μ*m/voxel studies of large plugs with diameters of 10–50 mm, or, alternatively, high-resolution, i.e. 1–10 *μ*m/voxel studies of smaller plugs with diameters of 1–10 mm^1–6^.

Laboratory measurements of a rock’s porosity and permeability are routinely performed on plugs having a diameter of 25 mm and height of 38.1 mm, respectively. This leads to a substantial gap, often more than 1000-fold, between the sample volumes that are imaged and probed in lab measurements, respectively. The difference in scales complicates the comparison between the porosity and permeability values obtained from direct petrophysical measurements with those indirectly measured from *μ*CT images. Such an analysis can be performed for spatially homogeneous rock samples such as sandstones^7^, however, it might fail for rather inhomogeneous rock samples, such as carbonates.

In the case of *μ*CT studies, the porosity and permeability measurements are computed from the generated 3D volumes either through calculation of the void space or through fluid simulations. To distinguish these measurements from the petrophysical characterization, we refer to these calculated porosity values as “computed” values and the direct petrophysical characterization as “laboratory” measurements.

In this work, we report full-scale, microscopically resolved X-ray tomographies of rock samples having the shape of a cylindrical plug with a diameter of 9.5 mm and a height of 25.4 mm. Each rock tomography is augmented by porosity and permeability values which were independently measured on the same rock samples in the lab. All rock samples were imaged and analyzed by following the same data acquisition protocol and by using the same equipment.

**Fig. 1:**
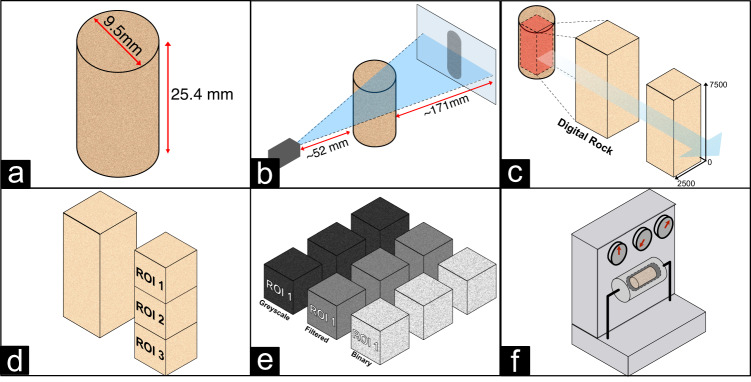
Conceptual overview of the rock sample study. (**a**) Schematic of a cylindrical rock plug sample having a length of 25.4 mm and a diameter of 9.5 mm. (**b**) Schematic of the X-Ray *μ*CT imaging process. (**c**) Visualization of the image cube cropping process. (**d**) Data cube subdivision by regions of interest (ROI). (**e**) Data cube processing from greyscale to binary images. (**f**) Schematic representation of porosity and permeability measurements in the lab.

Figure 1 illustrates a schematic overview of the steps followed in this study to produce the digital rock tomography dataset. As depicted in Fig. 1b–e, for each rock sample in the dataset, the scanned image is provided in three different formats: a first image file in raw format of the largest inscribed parallelepiped within the plug, a second raw file where the original image is cut to conform to a standardized parallelepiped of size 2500 × 2500 × 7500 voxels, and, lastly, a set of three 2500^3^ voxel cubes extracted from the standardized image and processed to binary. Finally, Fig. 1f illustrates the measurement of porosity and permeability of each sample in the lab. In the following section, we discuss the methods involved in image data acquisition and post-processing as well as the laboratory measurement techniques for obtaining porosity and permeability values.

## Methods

### Rock plug sample description

The carbonate and sandstone rock plug samples (Kokurec Industries Inc.) have a size of 9.5 mm diameter and 25.4 mm in length, as shown in Fig. 1a. The sample size was chosen for enabling full scale imaging with high resolution and petrophysical characterization on the same sample. Table 1 lists all rock samples analyzed in this work.

**Table 1:** List of rock samples analyzed in this study.

Sample Name	Rock	Type
1B	Silurian Dolomite	Carbonate
1C	Silurian Dolomite	Carbonate
4A	Indiana Limestone	Carbonate
5A	Lueders	Carbonate
6A	Mt. Gambier	Carbonate
13A	Castlegate	Sandstone
14A	Carbon Tan	Sandstone
15A	Bentheimer	Sandstone
18A	Liver Rock	Sandstone
20A	Idaho Gray	Sandstone
SD	Silurian Dolomite	Carbonate
I-151016	Indiana Limestone	Carbonate
GD	Guelph Dolomite	Carbonate
EdY	Edwards Yellow	Carbonate
EdW	Edwards White	Carbonate
EdB-1	Edwards Brown	Carbonate
DP	Desert Pink	Carbonate
2-ILC	Indiana Limestone	Carbonate

### Rock sample imaging and tomography

**Fig. 2:**
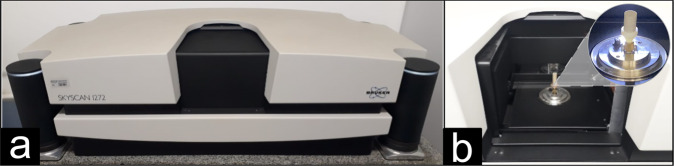
Experimental setup for rock micro-tomography. (**a**) X-Ray *μ*CT System. (**b**) Rotational sample stage with mounted rock plug sample.

We have acquired digital 3D image volumes from all samples in Table 1 using the X-ray *μ*CT system (Skyscan 1272, Bruker) shown in Fig. 2a. During image acquisition, the *μ*CT system produces a series of two-dimensional projections of the porous rock that are computationally transformed into 3D digital representation. Figure 2b shows the cylindrical rock plug vertically placed in the *μ*CT system.

We configured the image acquisition software (SkyScan1272 Control Program, version 1.2.0.0; Bruker) as follows: *I* = *100* *μA; V* = *100* *kV; Frame Averaging* = *3; Cu 0.11* *mm filter; Pixel Size* = *2.25* *μ*m*; Rotation of 360 with 0.1 steps; random movement range* = *2 to 4*. Table 2 lists the exact random movement parameters used for each sample each sample.

**Table 2:** Random movement parameter used on each sample.

Random Movement	
1C	2
1B	2
4A	3
5A	3
6A	3
13A	3
14A	3
15A	3
18A	3
20A	3
SD	4
I15	4
GD	4
EdY	3
EdW	3
EdB	4
DP	3
ILC	4

**Fig. 3:**
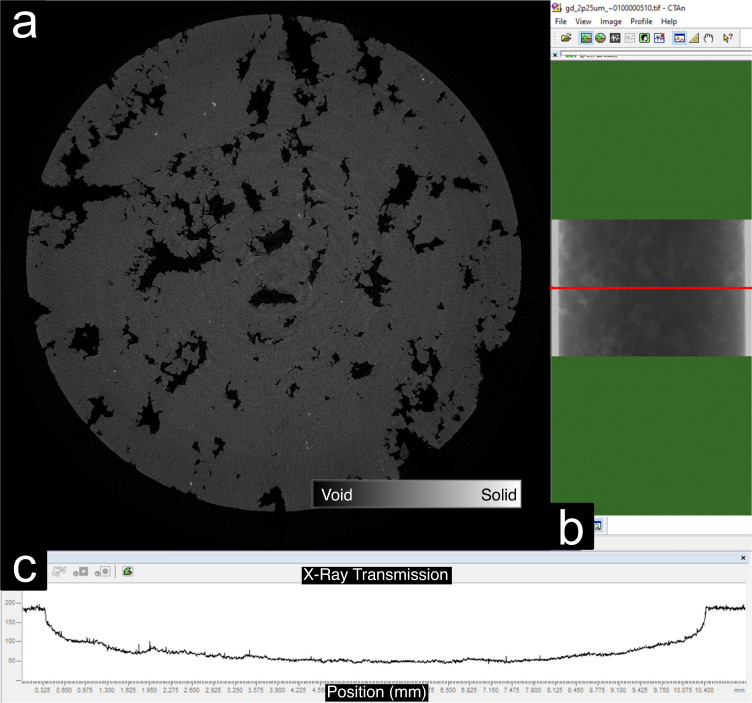
Analysis of X-ray attenuation through the sample. (**a**) Rock image taken close to the sample center where the darker regions represent the void spaces (**b**) User interface showing the cross-sectional intensity variations across the center of the sample (in red). (**c**) Intensity profile along the red line in (**b**) with a maximum and minimum signal around 200 and 50 grayscale levels, respectively.

To ensure a suitable sample size and gauge the X-Ray attenuation through the sample, we computed profile curves along the center of the plug. Figure 3a displays a center slice of a digital rock sample after reconstruction, Fig. 3b shows the data acquisition user interface indicating the height of the cross-sectional cutline across the center of the sample (in red), and Fig. 3c shows the transmission intensity profile for the sample GD (Guelph Dolomite). In this example, we observed that the transmission along the sample reaches a minimum grayscale level of around 50 at the sample center, with a maximum value of around 200. Samples with X-Ray transmission close to a 0 were discarded from the study.

### Rock image data processing workflow

After completion of image data acquisition, the reconstruction of the 3D image was performed by calculating the orthogonal slices from the radial projections using the Feldkamp algorithm^8^ implemented within the measurement system software (NRecon, version 1.7.4.6, with the Reconstruction engine InstaRecon, version 2.0.4.6, Bruker). In addition, the reconstruction involves the application of various data processing methods to reduce image artifacts generated by noise in the X-Ray signal during image acquisition. Such signal variations can occur due to fluctuations in the X-ray emission intensity, the detector sensitivity, or through attenuation of lower energy components within denser sample volumes.

The parameters for the reconstruction include Smoothing (using Gaussian kernel), Ring Artifacts Reduction and Beam-Hardening. We selected the most suitable configuration parameters by scanning the possible values with large steps of trial reconstructions, followed by fine tuning with smaller steps until the result was acceptable. We left the reconstruction histogram unchanged to cut and rescale it uniformly in subsequent steps of data processing. We defined the ROI such that it was contained inside the sample through all the slices. We left the undersample option unchecked as no digital binning was used in this study. All reconstruction settings for each sample can be found in the dataset, as described in the data records section.

**Fig. 4:**
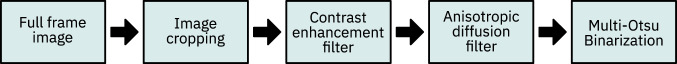
Rock data processing workflow applied to each image cube.

**Fig. 5:**
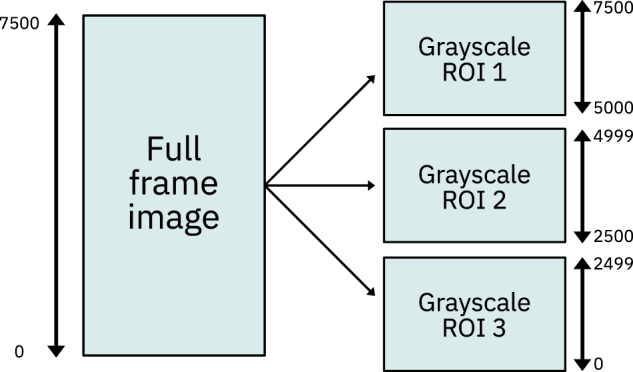
Splitting of the standardized image data volume into three regions of interest.

Once the 3D digital grayscale rock images were reconstructed, we applied the image data processing workflow outlined in Fig. 4 for removing measurement artifacts and separating the pore space from the rock matrix. In a first step, we cropped the full digitalized volume obtained from the *μ*CT measurements to a standard size of 2500 × 2500 × 7500 voxels. This way, the image data parallelepiped could be further split equally into three 2500^3^ voxel sized cubes for improved data handling, see Fig. 5.

In a next step, we applied a contrast enhancement filter to account for the varying mineralogic compositions of the samples studied for equalizing the contrast across all image data sets. The filter was applied to each 2500^3^ voxel volume independently, cutting off the histogram at the grayscale level in which the accumulated histogram achieved 99.8%, and mapping the remaining grayscale levels back to the [0, 255] interval, thus ensuring an efficient utilization of the entire gray level range.

In a next step, the image data was processed by an anisotropic diffusion filter implemented within the measurement system software (Bruker, version 1.20.8.0) for reducing image noise. The filter was set to 3D space, the type used was Privilege high contrast edges (Perona-Malik), the number of iterations set to 5 and the gradient threshold set to 10. The user defined integration constant option was left unchecked.

Finally, we evaluated both Multi-Otsu and Otsu methods^9,10^ for determining a grayscale threshold level for segmentation into solid and void spaces, leading to a binary cubic volume. We observed that a binary segmentation was not capable of properly discerning between matrix and pore structure for all samples studied, mainly due to sample sub-porosity, i.e. image regions of intermediary grayscale levels caused by heterogenous mineral composition, or limited pixel resolution. Therefore, a 3-level Otsu method was chosen.

**Fig. 6:**
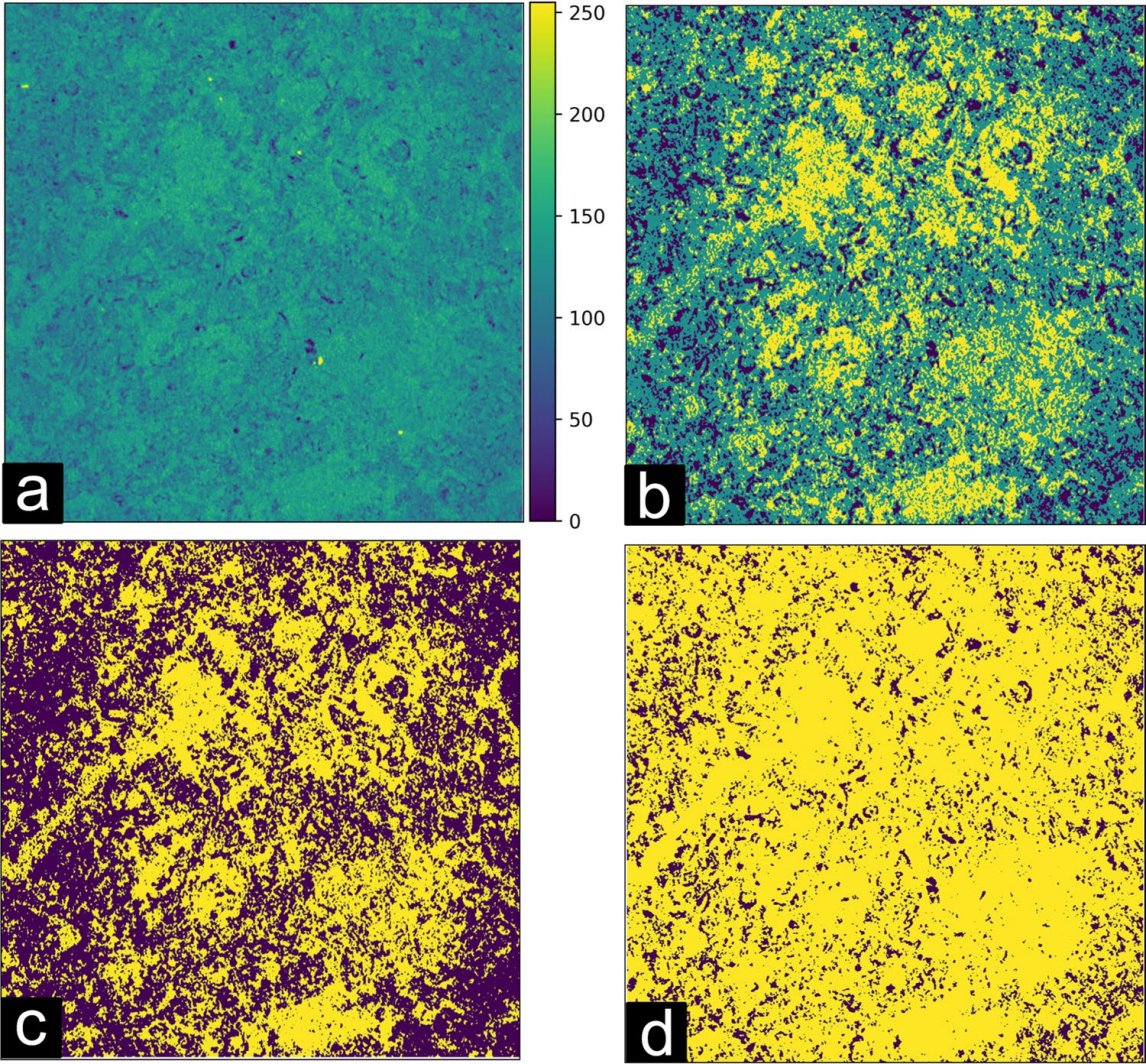
Effect of the segmentation algorithm on computed porosity. (**a**) Filtered grayscale image from sample 5A. The colorbar represents the grayscale level from 0 to 255. (**b**) Processed image segmented by means of the Multi-Otsu algorithm (n = 3) shows three distinct phases: the solid matrix in yellow, void space in black, and the intermediary class in green. (**c**) Segmented images using the Otsu algorithm and (**d**) Multi-Otsu method after merging the solid matrix and intermediary classes. Yellow represents the calculated solid matrix from merging the two classes and purple represents the void space. The side length of each image is 5.625 mm.

To ensure proper segmentation, the intermediary class identified by the Multi-Otsu algorithm (corresponding to the sub-porous region) was considered part of the mineral matrix. Figure 6 shows the effect on the digitalized rock image when applying the Multi-Otsu algorithm. Figure 6a displays the grayscale filtered image extracted from sample 5A after undergoing the various processing steps shown in Fig. 4. Figure 6b shows the same rock sample image after the Multi-Otsu algorithm has identified three different regions in this heterogeneous sample, a black region representing the pore space, a yellow area representing the rock matrix and, in green, the intermediary phase.

**Fig. 7:**
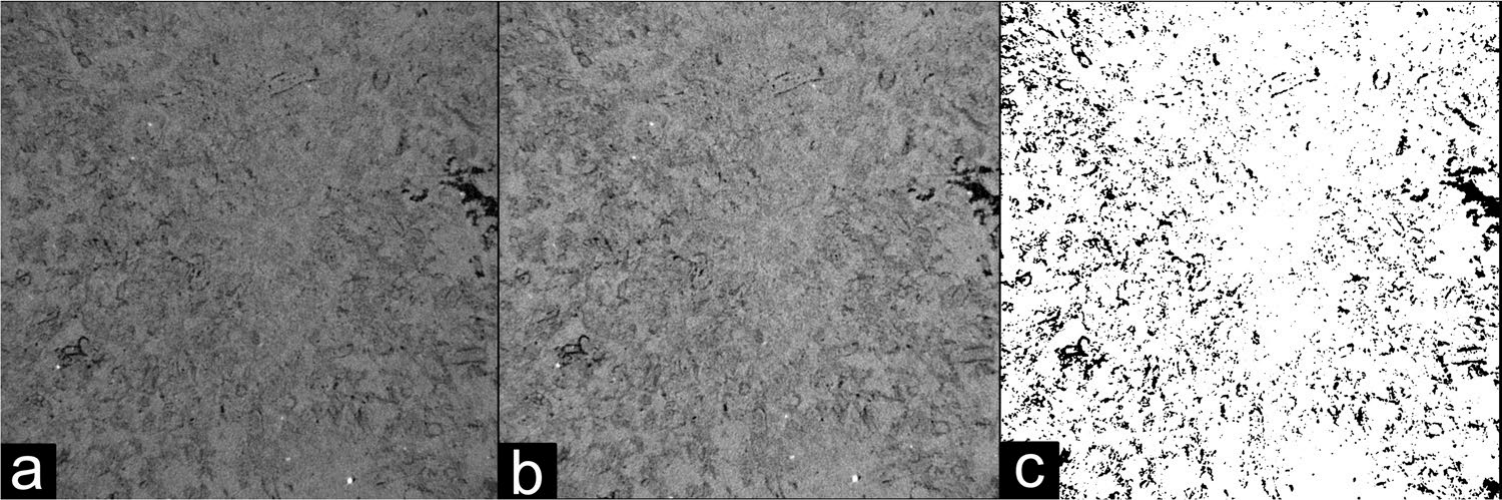
Representative example of the image processing end-to-end. Example image in (**a**) raw, (**b**) filtered and (**c**) segmented mode, representing each step in our image processing workflow. The side length of each image is 5.625 mm.

By calculating the ratio of void to solid space in the binarized volumes, we can estimate the porosity of the sample and compare it with the laboratory measurement value of 13.89%. We obtain a porosity of 33.68% with the 2-level Otsu method while the 3-level Multi-Otsu method provides 8.5% porosity (after merging two levels in the ROI 1 cube). Although this approach may lead to sub-estimation of porosities from the *μ*CT images, it helped to mitigate the limitations due to the lack of region contrast and produced more accurate porosity estimates across all samples. Despite these limitations, we expect that, due to the resolution limit of the X-Ray *μ*CT, porosity estimates based on the tomographic volumes yield values lower than those obtained in the petrophysical characterization, which seems compatible with our results. Table 3 lists for each sample the thresholds applied in this study. The cutoff point for binarization was defined as setting pixels equal or greater than the value of the threshold to 1. As a representative example of the effects of data processing, we show in Fig. 7 a single tomographic slice in raw, filtered, and binary formats, respectively.

**Table 3:** Computed thresholds used in the segmentation of each ROI.

Sample Name	ROI 1 Threshold	ROI 2 Threshold	ROI 3 Threshold
1B	93	93	98
1C	91	93	94
4A	94	95	97
5A	105	120	126
6A	79	79	81
13A	72	73	74
14A	100	102	103
15A	67	74	77
18A	52	54	59
20A	76	77	80
SD	83	83	85
I-151016	88	88	90
GD	93	93	93
EdY	88	89	84
EdW	106	121	124
EdB-1	64	68	66
DP	88	82	84
2-ILC	78	80	80

### Lab experimental characterization of petrophysical properties of rock samples

After image data acquisition, we measured porosity and absolute permeability of each rock sample at an overburden pressure of 500 psi in Nitrogen gas at 21 °C using standard equipment (UltraPore-300 and UltraPerm-600, Core Labs). We determined pore and solid volumes based on the known flow cell volume and overburden pressure by assuming isothermic conditions. We estimated the pore density from the ratio between the solid mass and volume. All petrophysical characterization methods were performed following API RP 40 best practices for core analysis^11^. The experimental porosity and permeability values are provided in Table 4.

**Table 4:** Porosity and permeability values for each rock sample analysed in this study.

Sample Name	Laboratory Porosity (%)	Computed Porosity				Laboratory Permeability (mD)	
		ROI 1	ROI 2	ROI 3	Mean (%)	Air	Klinkenberg
1B	8.22	0.13	0.12	0.11	11.70	67.39	61.34
1C	13.46	0.15	0.13	0.13	13.34	373.14	353.53
4A	14.71	0.08	0.08	0.08	8.27	23.11	20.41
5A	13.89	0.08	0.13	0.12	11.23	55.79	50.66
6A	53.45	0.38	0.40	0.39	39.18	144.41	134.37
13A	27.39	0.17	0.17	0.17	17.23	638.56	610.16
14A	23.49	0.13	0.13	0.13	13.16	67.56	61.53
15A	22.91	0.22	0.21	0.21	21.29	0.42	0.31
18A	25.00	0.21	0.21	0.22	21.44	392.97	372.64
20A	28.48	0.28	0.28	0.27	27.54	>5,000.00	5,000.00
SD	14.67	0.14	0.13	0.13	13.19	80.59	73.80
I-151016	17.57	0.13	0.13	0.11	12.40	50.11	45.23
GD	14.35	0.11	0.12	0.12	11.45	1228.80	1184.53
EdY	25.96	0.16	0.17	0.17	16.84	11.45	9.80
EdW	15.38	0.12	0.12	0.15	12.73	0.63	0.48
EdB-1	29.63	0.20	0.16	0.19	18.45	14.61	12.59
DP	24.54	0.18	0.19	0.19	18.61	47.98	43.26
2-ILC	17.05	0.15	0.14	0.13	14.03	72.94	66.57

## Data Records

**Fig. 8:**
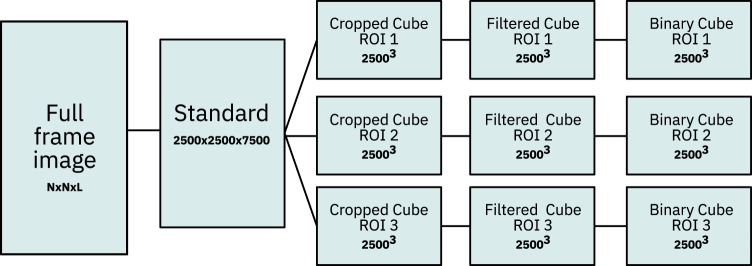
Overview of the dataset file structure.

The dataset^12^ is provided in five different volume types and formats for each sample, as summarized in Fig. 8. The suffix inside the parenthesis designates the naming scheme used for the dataset files:

- **Full Frame (_grayscale_full):** Data obtained from the reconstruction of the *μ*CT projections. During reconstruction, the volume edges are removed, however, the largest inscribed parallelepiped within the plug is retained, thus leading to different sized parallelepipeds.
- **Standard (_grayscale_standard):** Volume cropped into a standard size of 2500 × 2500 × 7500 voxels.
- **Cropped cubes (_grayscale_ROI-X):** 2500^3^ voxel cubes extracted from the standard volume. The X designates the number of the cube, with values ranging from 1 to 3, cut top-down from the parallelepiped.
- **Filtered cubes (_grayscale_filtered_ROI-X):** Data obtained from the grayscale cubes through the application of contrast enhancement and noise reduction filters. The X designates the number of the cube, with values ranging from 1 to 3, cut top-down from the parallelepiped.
- **Binarized cubes (_binary_ROI-X):** Binary image data obtained from the filtered grayscale cubes. Each grayscale cube was segmented at a threshold level calculated using the Multi-Otsu algorithm with a number of classes set to three (see Table 3). The X designates the number of the cube, with values ranging from 1 to 3, cut top-down from the parallelepiped.

In addition to the above, we provided as supporting information:

- **HDR file:** File containing the cube size information for each sample.
- **Dataset_Information.xlsx:** File containing the naming convention used for all files as well as possible name changes that may happen during file decompression. This file also includes all acquisition and reconstruction parameters for each of the measurements encompassed in this work.
- **qrm_10w__ir_rec_tra_X.raw:** Data obtained from a standard microCT Bar pattern (NanoPhantom, QRM) for estimation of the spatial resolution of the microCT measurements. The X designates the reference targed imaged, either horizontal or vertical.

The dataset^12^ acquired in this study and reported in the manuscript is available under the 10.25452/figshare.plus.21375565.v6.

## Technical Validation

### Comparison between computed and laboratory porosities

We now compare the porosity *p* computed based on the rock image data (with binary voxels *b*_*i,j,k*_ values 0 and 1 for void and solid matrix spaces, respectively) with the porosities measured following standard petrophysical lab methodology. For each ROI cube, we computed the porosity based on Eq. 1:

1 $$p=1-\frac{1}{{N}^{3}}\mathop{\sum }\limits_{i=0}^{N}\mathop{\sum }\limits_{j=0}^{N}\mathop{\sum }\limits_{k=0}^{N}{b}_{i,j,k}$$

Where the mean porosity value of each sample was calculated by averaging the solid fraction value obtained for each region of interest.

**Fig. 9:**
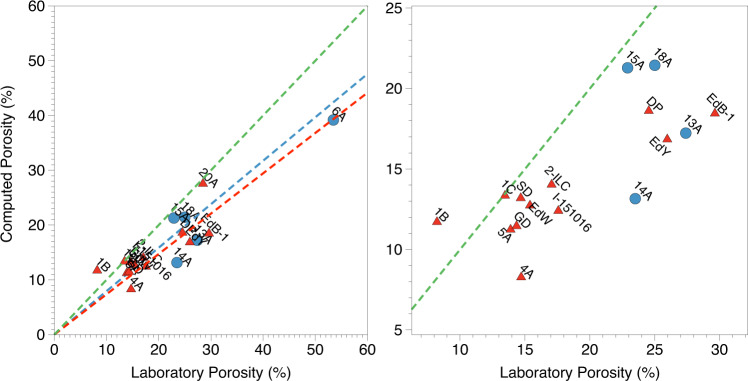
Analysis of computed and laboratory porosities for sandstone (blue) and carbonate (red) samples. The green line represents identity. The blue (*R*^2^ = 0.243) and red (*R*^2^ = 0.889) lines represent linear fits to the carbonate and sandstone data, respectively. The panel on the right represents a zoom of the plot on the left.

Figure 9 compares the computational (averaged between all three ROIs) and laboratory porosity results for all samples in the dataset. As expected, except for sample 1B, all samples are located close to or below the green line due to under-estimation of porosity, most probably caused by limitations in image resolution. Overall, we find that the image-based method provides robust porosity estimates for both sandstone and carbonate samples. Future research work is needed to connect the porosity and permeability values for each sample based on image analysis. To that end, we believe that the data published in this study provides key contributions.

### Limitations

#### Pore space estimation

The estimation of the pore network geometry and porosity strongly depends on the methods used to translate the raw tomography data into a binarized (i.e. void and pore space) volume for flow simulations. As discussed in the image processing workflow section, different image processing algorithms may lead to different binarized volumes which, consequently, will affect the computed porosity values and permeability measurements derived from flow simulations. One of the factors affecting the binarization of the tomography data is the presence of porous regions with pore sizes smaller than or close to the measurement resolution, referred to as sub-porous regions. These areas will show lower contrast when compared with the void spaces, and thus make it harder to properly segment and characterize the pore structure by misrepresentation of the rock matrix.

The Multi-Otsu algorithm was chosen due to leading to better agreement with laboratory porosity while making sure that the computed porosity results were lower than the one obtained in the petrophysical characterization. This assumption comes from the fact that the petrophysical characterization is expected to have a higher resolution than the microCT measurements. Our approach, however, can lead to outliers where the computed porosity values are greater than the laboratory porosity, as shown in Fig. 9. This discrepancy is likely caused by the image segmentation step.

**Fig. 10:**
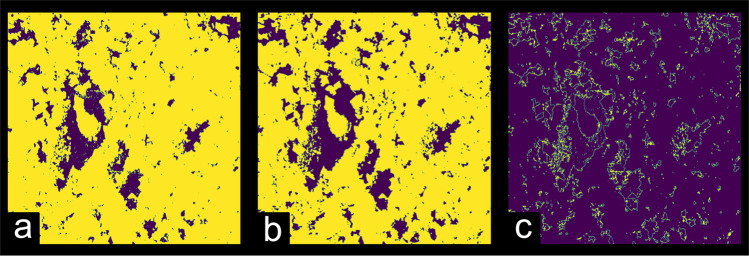
Effect of the segmentation algorithm to the computed porosity. (**a**) skimage.filters.threshold_triangle algorithm. (**b**) skimage.filters.threshold_multiotsu algorithm. (**c**) Calculated image subtraction from images a and b (triangle - multiotsu). The side length of each image is 5.625 mm.

To exemplify, we have recalculated sample 1B’s porosity using the triangle segmentation algorithm^13^ and obtained a lower computed porosity than both the Multi-Otsu and Otsu algorithms, as shown in Fig. 10. In this case, the computed porosity from the triangle algorithm is lower than the value obtained from the petrophysical characterization (8.2%), as shown in Table 5.

**Table 5:** Effect of the segmentation algorithm to the computed porosity.

Sample	Computed Porosity				Laboratory Porosity (%)	Threshold Algorithm	Segmentation Threshold		
	ROI 1	ROI 2	ROI 3	Mean (%)			ROI 1	ROI 2	ROI 3
1B	0.063	0.057	0.046	5.6	8.2	threshold triangle	10	10	11
1B	0.138	0.129	0.118	12.8	8.2	threshold otsu	110	111	113
1B	0.127	0.117	0.108	11.7	8.2	threshold multiotsu	93	93	98

Our choice of prioritizing consistency leads to a compromise, as some samples might benefit from different segmentation algorithms. It is conceivable that an in-depth exploration of the proper segmentation algorithm for each rock type or sample could yield a greater agreement with the petrophysical measurements.

Although the binary images in our Dataset are presented for the convenience of the end-user, they are not by any means the most complete representation of the sample. We have included the raw grayscale images to allow users looking for more robust analysis to test different processing and segmentation algorithms that may lead to a better representation of the pore network structure than the ones presented in this work.

#### Image spatial resolution

We have conducted supporting measurements of a standard MicroCT bar pattern (BarPattern NANO V2, QRM) to directly estimate horizontal and vertical spatial resolution. The source power was kept at 10 W as during the rock sample acquisition, but different filter, current, and voltage settings were used to account for the sample transparency (please refer to the “Dataset_Information.xlsx” file for further information).

**Fig. 11:**
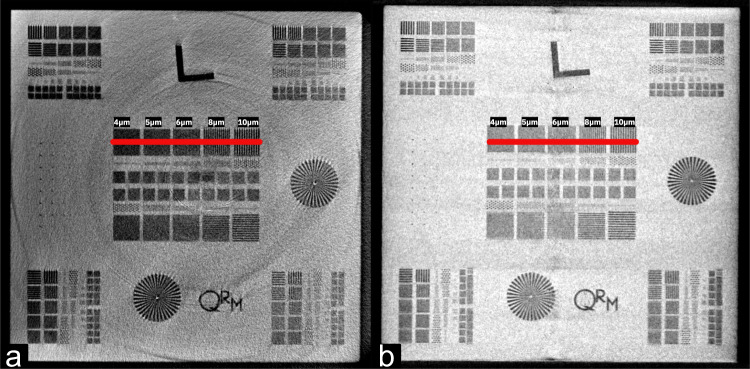
MicroCT bar pattern reference measurement. Images showing the horizontal (**a**) and vertical (**b**) bar patterns from which the cross sections were measured for estimation of the spatial resolution. The side length of each target is 3 mm.

**Fig. 12:**
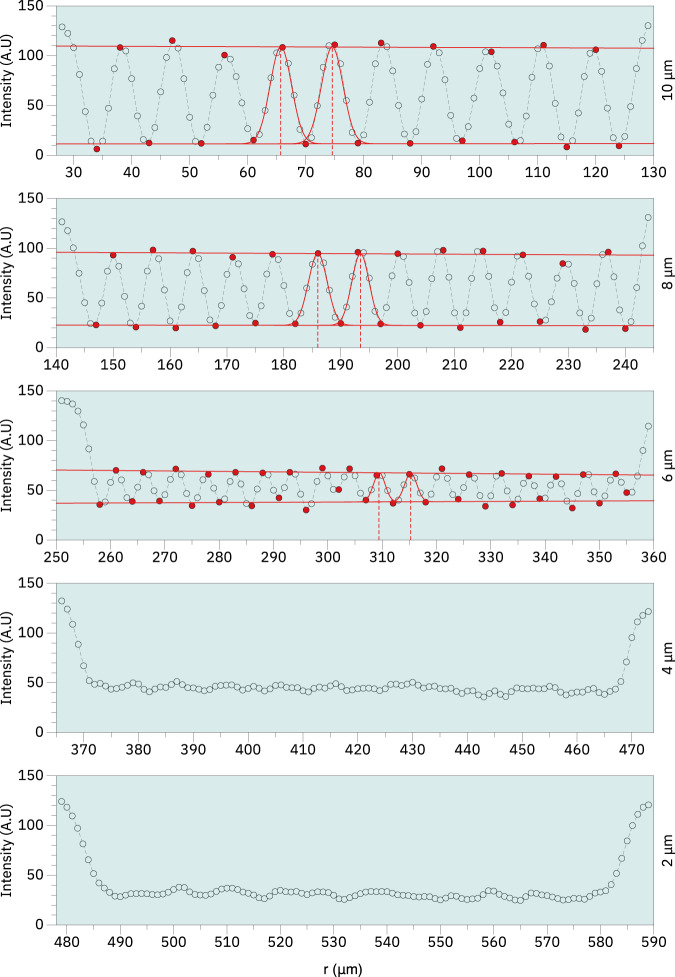
Image cross section of reference region 1A obtained from the horizontal bar pattern reference. Measurement of the cross sections obtained from the bar pattern reference with linewidths between 10-2 *μ*m. Image contrast was estimated according to Eq. 2 by calculating a linear fit passing through the maximum and minimum points shown in red. The calculated contrast values were approximately 0.82, 0.61 and 0.44 respectively for 10 *μ*m, 8 *μ*m and 6 *μ*m. Peak distance was obtained from fitting a Gaussian curve in subsequent peaks and calculating the distances between the curve centers. The estimated peak distances were approximately 9 *μ*m, 8 *μ*m and 6 *μ*m for 10 *μ*m, 8 *μ*m, and 6 *μ*m lines respectively. Neither contrast nor peak distance was calculated for 4 *μ*m and 2 *μ*m due to the profiles not showing well resolved peaks.

**Fig. 13:**
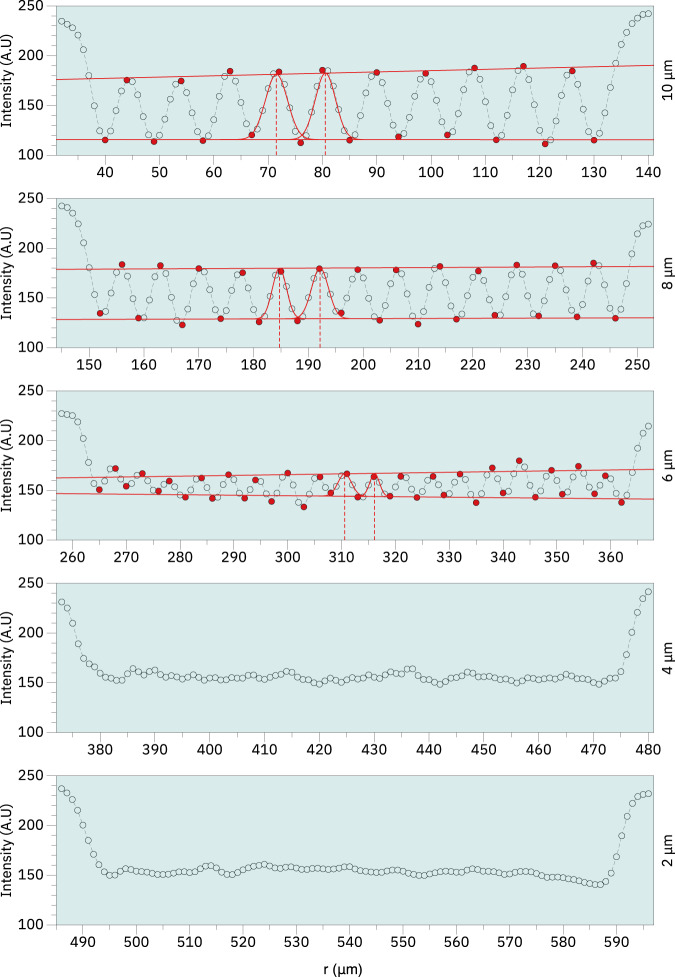
Image cross section of reference region 1A obtained from the vertical bar pattern reference. Measurement of the cross sections obtained from the bar pattern reference with linewidths between 10-2 *μ*m. Image contrast was estimated according to Eq. 2 by calculating a linear fit passing through the maximum and minimum points shown in red. The calculated contrast values were approximately 0.22, 0.17 and 0.07 respectively for 10 *μ*m, 8 *μ*m and 6 *μ*m. Peak distance was obtained from fitting a Gaussian curve in subsequent peaks and calculating the distances between the curve centers. The estimated peak distances were approximately 9 *μ*m, 7 *μ*m and 6 *μ*m for 10 *μ*m, 8 *μ*m, and 6 *μ*m lines respectively. Neither contrast nor peak distance was calculated for 4 *μ*m and 2 *μ*m due to the profiles not showing well resolved peaks.

Figure 11 shows the horizontal and vertical bar pattern targets from which the cross sections presented in Figs. 12, 13 were derived for estimation of the spatial resolution. In both cases we have examined the region along the red line (target region 1A) shown in Fig. 11 as the line width range covers the nominal microCT resolution of 5 *μ*m. We estimate that for both vertical and horizontal measurements, the spatial resolution falls between 5–6 *μ*m or roughly twice the image pixel size. In both cases the image contrast was calculated using the Michelson contrast definition^14^ according to Eq. 2:

2 $$Contrast=\frac{Max-Min}{Max+Min}$$

Where the maximum and minimum values were estimated from a linear regression obtained from the maximum and minimum values of the line pattern signal along the curve, respectively.

We have added the reconstructed measurements obtained from the reference target to the Dataset. We hope these data will enable users looking for an alternative characterization of the spatial resolution to conduct their own validation.

## Data Availability

The algorithms used for processing and segmenting the raw grayscale images are available as Python code at: https://github.com/IBM/microCT-Dataset. The code repository contains Jupyter Notebooks for simplifying data processing and visualization along with usage guidance.
